# When Chest Pain Isn’t What It Seems: A Case of Cardiac Lymphoma

**DOI:** 10.7759/cureus.78267

**Published:** 2025-01-30

**Authors:** Ahmed M Basuoni, Waleed D A, Airton Leonardo de Oliveira Manoel, Asma Atfeesh

**Affiliations:** 1 Cardio-Oncology, Sultan Qaboos Cancer Care and Research Center, Muscat, OMN; 2 Cardiology, Sultan Qaboos Cancer Care and Research Center, Muscat, OMN; 3 Trauma Intensive Care Unit, Hamad General Hospital, Doha, QAT; 4 Hematology, Sultan Qaboos Comprehensive Cancer Care and Research Center (SQCCCRC) University Medical City, Muscat, OMN

**Keywords:** acute coronary syndrome, cardiovascular magnetic resonance (cmr), echocardiography, positron emission tomography (pet), primary cardiac lymphoma

## Abstract

Primary cardiac lymphoma (PCL) is an exceedingly rare malignancy, often presenting with heart failure or arrhythmias. This case report describes a 61-year-old male with a history of diabetes, hypertension, and ischemic heart disease who presented with symptoms mimicking acute coronary syndrome (ACS). Despite significant stenosis in the left anterior descending artery (LAD), advanced imaging, including cardiovascular magnetic resonance (CMR) and positron emission tomography (PET) scan, revealed a large infiltrative mass consistent with PCL. The patient was diagnosed with high-grade B-cell lymphoma but unfortunately succumbed to rapid disease progression before initiating chemotherapy. This case emphasizes the importance of considering alternative diagnoses in atypical ACS presentations and highlights the critical role of multimodal imaging in accurately diagnosing rare cardiac tumors. Early diagnosis and prompt treatment are essential for improving outcomes in PCL, an aggressive malignancy with limited treatment options. The case also underscores the need for heightened clinical awareness and further research into the pathophysiology and management of PCL.

## Introduction

Primary cardiac tumors are exceedingly rare, with an incidence of less than 0.1%. In contrast, metastatic cardiac involvement is significantly more common, occurring over 20 times as frequently. Autopsy studies have reported cardiac metastases in up to 20% of patients who passed away from cancer [1].

Cardiac tumors may be symptomatic or can be diagnosed accidentally during evaluation for a seemingly unrelated problem or unusual presentation [2]. Primary cardiac lymphoma (PCL) is a very rare disorder. According to the literature, only 40 cases were identified between 1995 and 2002, with outcomes generally reported as poor [3].

In prior studies, PCL has most commonly presented with symptoms of congestive heart failure (CHF) or arrhythmias. However, cases presenting with acute chest pain resembling acute coronary syndrome (ACS) have been less frequently documented. This report highlights a case of PCL with a rapid and aggressive progression, initially manifesting as acute chest pain mimicking both ACS and acute aortic syndrome (AAS) [4].

## Case presentation

**Figure 1:**
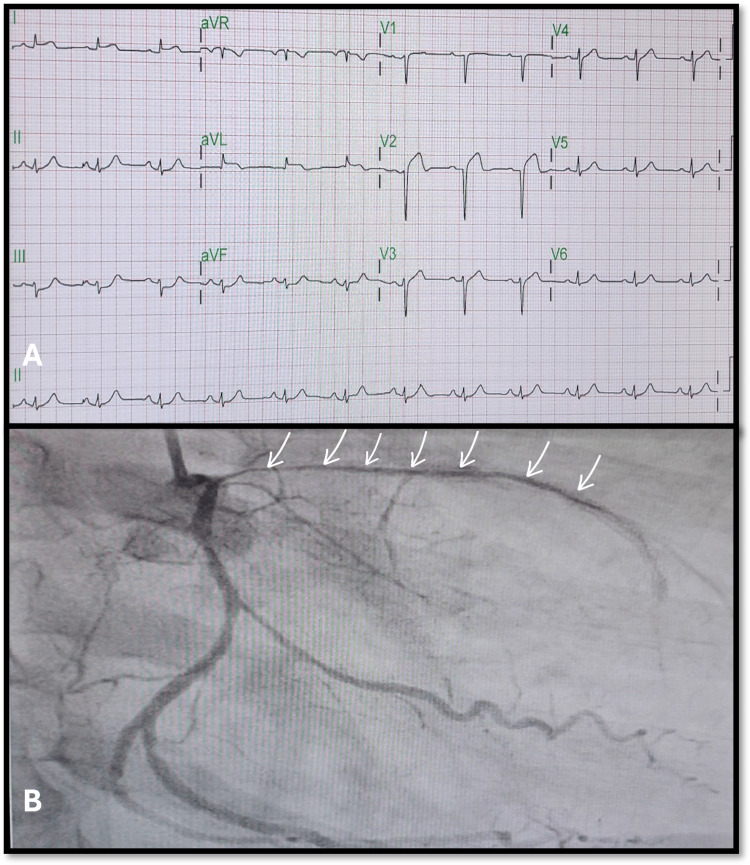
Electrocardiographic and coronary angiographic findings on presentation (A) ECG on presentation displayed ST elevation in the anterior and anterolateral walls with reciprocal ST depression. (B) Coronary angiography during the current presentation revealed diffuse stenosis from the distal left main, encompassing the entire course of the LAD (multiple white arrows).

A 61-year-old male patient is known to have diabetes mellitus, hypertension, and ischemic heart disease. He had a history of percutaneous intervention (PCI) with two drug-eluting stents (DES) in a left anterior descending artery (LAD). He presented to the emergency room with anterior wall myocardial infarction (AWMI) (Figure 1A). Coronary angiography showed diffuse stenosis in LAD (Figure 1B). No intervention was done due to the high suspicion of mass compressing the whole course of LAD.

**Figure 2:**
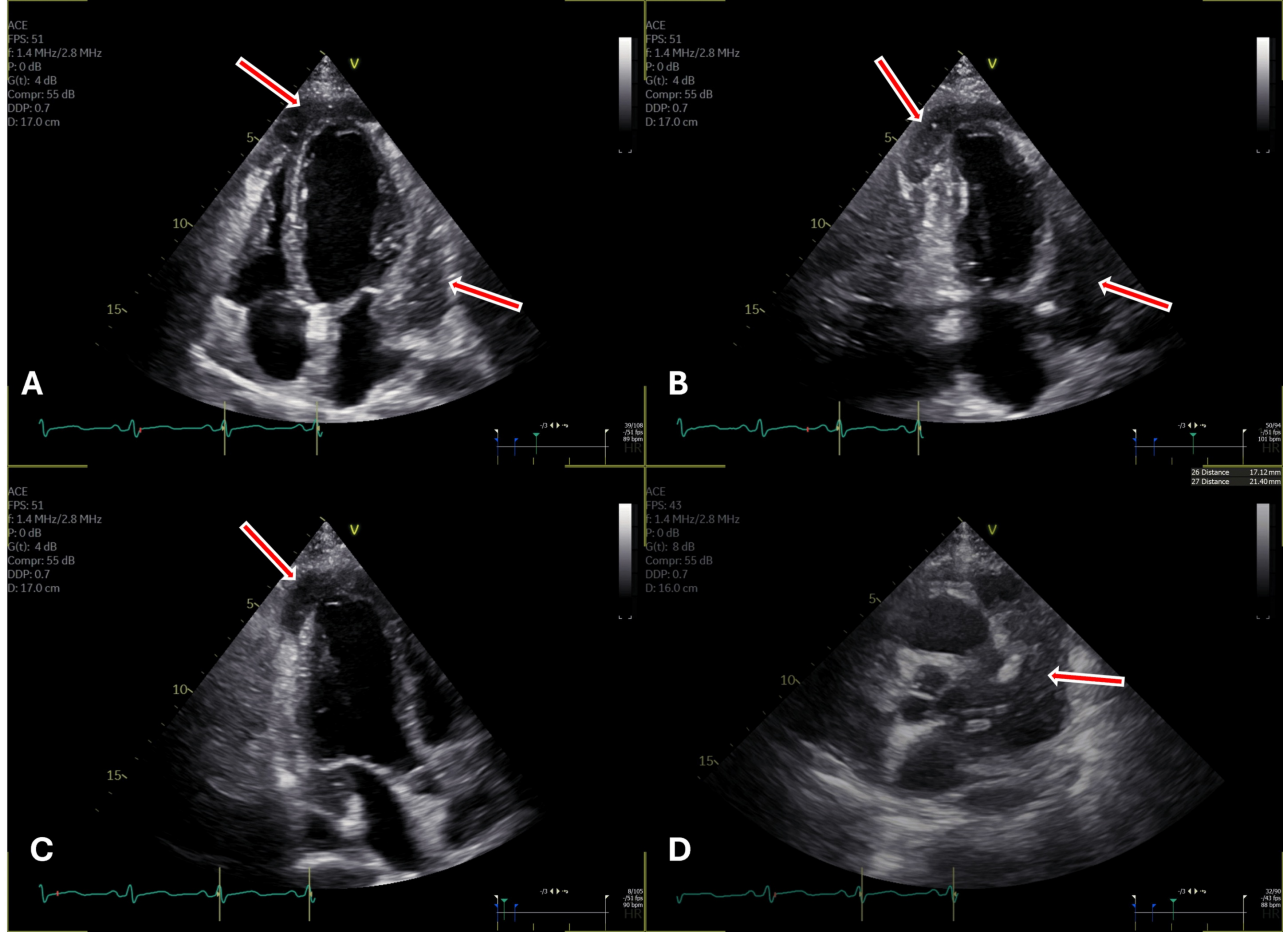
Multiplanar echocardiographic views demonstrating a suspected mass surrounding the left ventricle. (A) Four-chamber view illustrating the suspected mass surrounding the left ventricle (LV) and extending to the apex (arrows). (B) Two-chamber view illustrating the suspected mass surrounding the left ventricle (LV) and extending to the apex (arrows). (C) Three-chamber view illustrating the suspected mass surrounding the left ventricle (LV) and extending to the apex (arrows). (D) Short axis at great vessels level view illustrating the suspected mass surrounding the left ventricle extending to the pericardium (arrows).

Echocardiography showed regional wall motion abnormalities (RWMA) in the anterior wall and anterolateral wall with a high suspicion of mass surrounding the left ventricle extended to apex (Figures 2A-2D).

**Figure 3:**
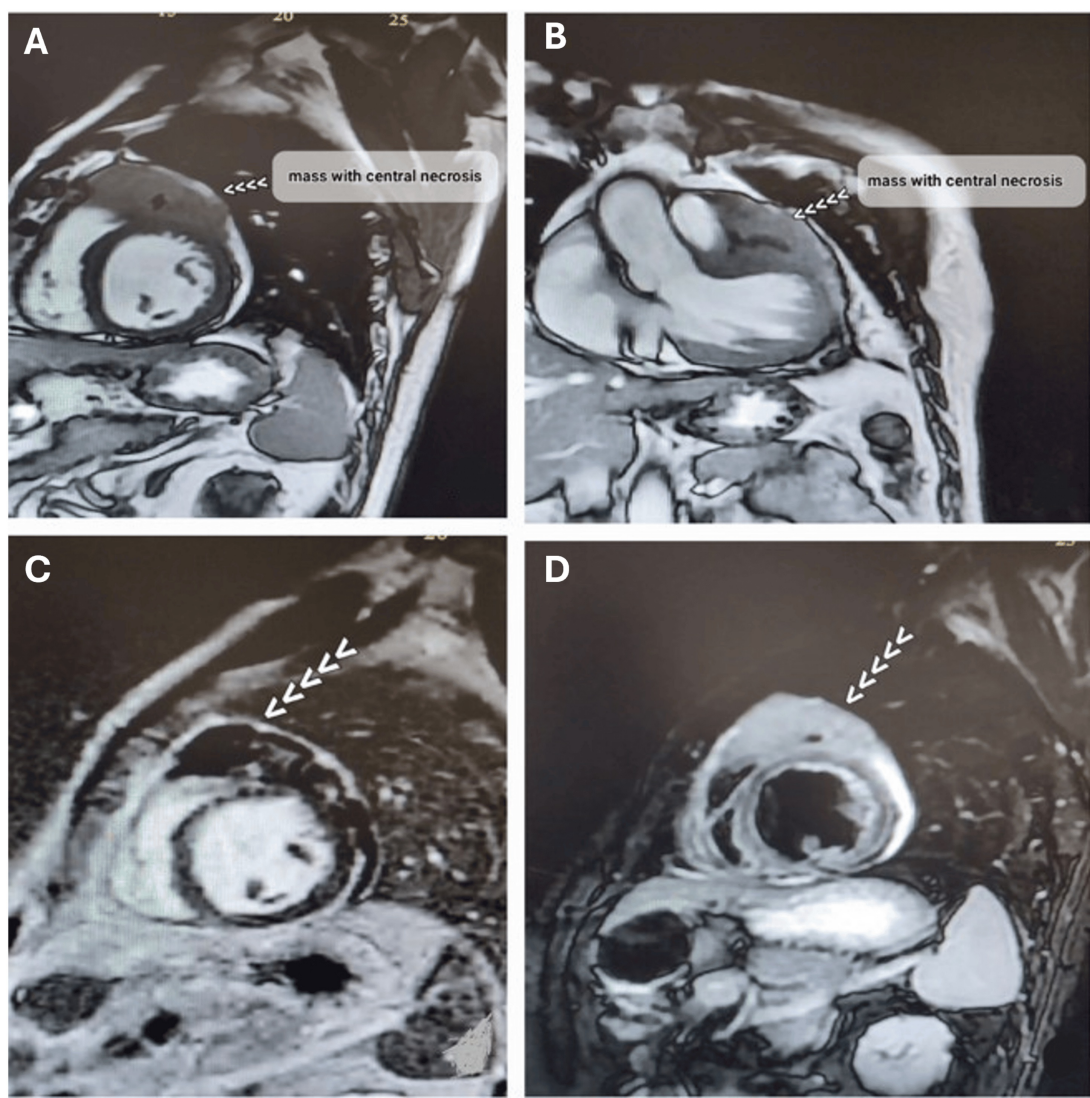
Cardiac MRI sequences for tissue characterization (A) Cine sequence of a short-axis view showing a mass surrounding the anterior wall with central necrosis (arrow). (B) Cine sequence of the left ventricular outflow tract (LVOT) view illustrating the mass surrounding the anterior wall with central necrosis (white arrows). (C) Late Gadolinium enhancement sequence showing uptake by the mass (pointed by white arrows). (D) Hyperintense in T2 sequence (pointed by white arrows).

CMR showed a large enhancing and infiltrative mass with central necrosis related to the anterior wall of the left ventricle, which after several sequences in CMR, the differential diagnosis was either Lymphoma or angiosarcoma (Figures 3A-3D).

**Figure 4:**
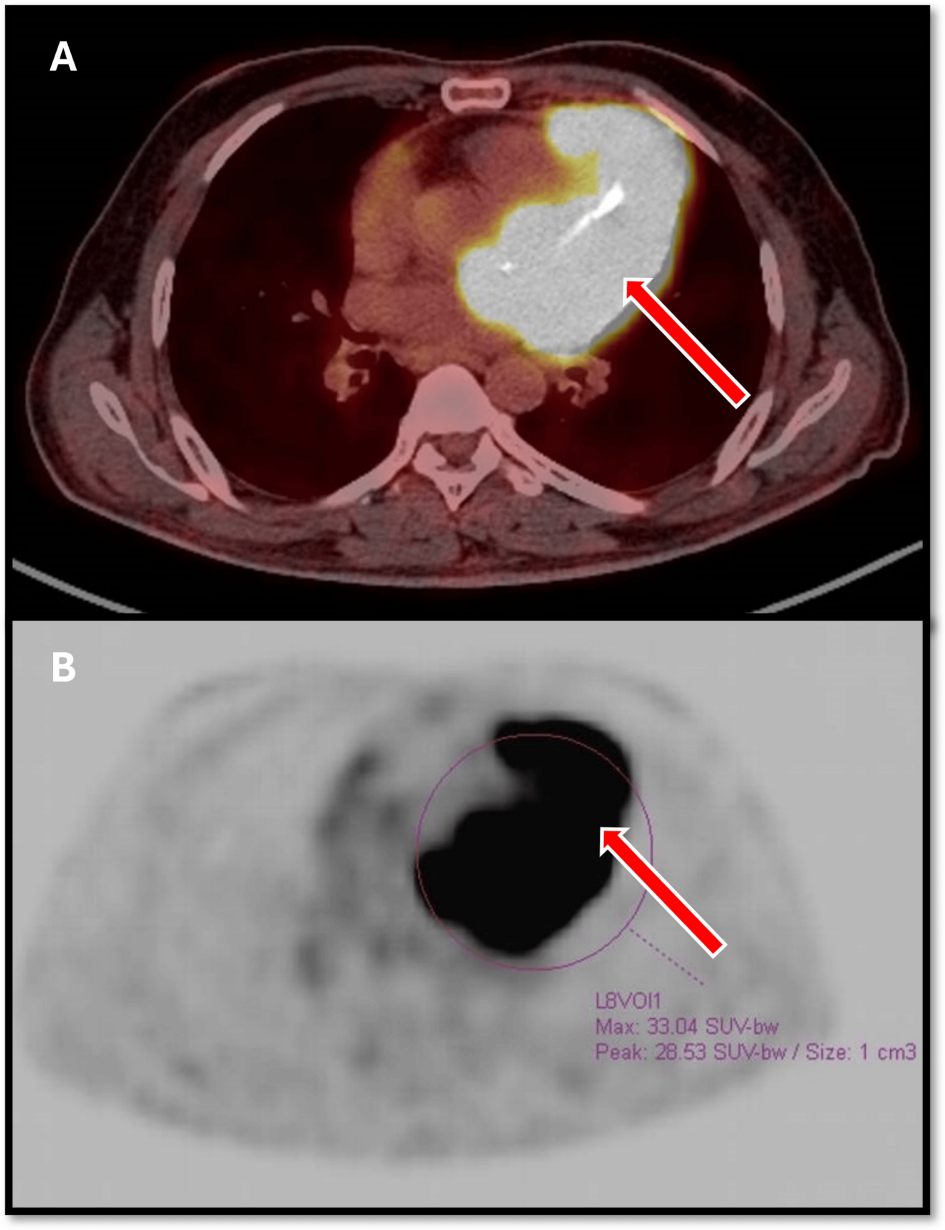
Positron emission tomography (PET) findings (A, B) Heterogeneously intense infiltrating mass in the left ventricle highly suspicious of malignancy, with possible pericardial involvement.

The patient was shifted immediately to our oncology center for further investigations. A positron emission tomography (PET) scan showed a heterogeneously very intensified infiltrating mass of the left ventricle highly suspicious of malignancy, with possible pericardial involvement (Figures 4A, 4B).

He underwent a computed tomography (CT) guided biopsy where the iliac lesion showed that the neoplastic cells are positive for high-grade B-cell lymphoma, not otherwise specified (NOS). Unfortunately, during the initiation of high dose steroids as per protocol and prior starting of Rituximab, Cyclophosphamide, Hydroxydaunomycin (Doxorubicin), Oncovin (Vincristine), and Prednisone (RCHOP) chemotherapy the patient developed sudden severe chest pain and ventricular tachycardia (VT) which had been treated by Amiodarone infusion and defibrillator followed by cardiac arrest. CPR started and the patient passed away.

## Discussion

This case initially showed clinical manifestation mimicking ACS. However, the patient had a history of a previous myocardial infarction. The recurrent visits to the emergency department with chest pain, elevated cardiac enzymes, and suspicions from echocardiography and coronary angiography of something else causing diffuse stenosis throughout the entire artery led to the consideration of alternative diagnostic modalities for a more accurate diagnosis.

This suggests that the standard diagnostic approaches for ACS might not be fully elucidating the underlying issue in this case. The need for alternative modalities could include imaging techniques or diagnostic tests beyond the usual ones employed for ACS to identify the specific cause of diffuse stenosis in the entire artery. This could involve exploring other cardiovascular or systemic conditions that may mimic ACS but require a different diagnostic and therapeutic approach.

Cardiovascular magnetic resonance (CMR) has become the gold standard for non-invasive evaluation of the heart, owing to its safety, high inter-observer consistency, quantitative accuracy, and capability to characterize myocardial tissue. CMR is also considered a gold standard tool for diagnosing other cardiac causes of ACS [5].

The diagnosis of PCL in this case was incidental. Clinical presentations mimicking ACS are extremely rare among previously reported PCL cases [6]. In our case, the presentation resembled ACS, with symptoms, ECG changes, and elevated troponin levels, likely attributed to myocardial and pericardial infiltration by PCL. Although significant diffuse stenosis was observed in the LAD artery, the nature of the chest pain, cardiac marker patterns, and ECG findings remained unchanged after admission. This suggests that the clinical features were more consistent with PCL infiltration or external compression, rather than ACS caused by LAD stenosis. The likely cause of death in this case was either external compression by the lymphoma, coronary artery infiltration, or myocardial infiltration [4].

It is reported in the literature that PCL predominantly affects the right side of the heart and is often presented with CHF and arrhythmias. Atrial arrhythmias and atrioventricular (AV) block are the most observed arrhythmias in these cases. However, VT is rarely reported, likely because many patients with VT die suddenly before a definitive diagnosis of PCL can be made [3,4,6].

In our case, the patient initially presented with symptoms mimicking ACS, which led to a delay in diagnosis and allowed the tumor to progress and become more aggressive. A stent was placed during the first presentation, and the patient survived for approximately six months. However, recurrent ST elevation occurred, reinforcing our view that treating the underlying cause is the only definitive approach. Early diagnosis and timely initiation of lymphoma-directed therapy, including steroids, are crucial in such cases. Another significant challenge was the tumor's unusual involvement of the left side of the heart, which not only contributed to ischemia but also predisposed the patient to malignant ventricular arrhythmias-further complicating management and being associated with a high mortality rate [6].

Diffuse large B cell lymphoma (DLBCL) is the most common type, making up about 30% of all lymphomas. DLBCL is a fast-growing, aggressive form of NHL. DLBCL is fatal if left untreated but with timely and appropriate treatment [7]. PCL can be diagnosed early because of advances in cardiac imaging. As cardiac lymphoma can be fatal in a few weeks, rapid diagnosis and treatment are necessary to save the patients [4,7]. Using multiple cardiac imaging modalities can enhance the accurate diagnosis of many unusual presentations of ACS, especially due to their powerful capability in tissue characterization [8,9].

Our case report shows the unusual presentation of ACS, rare primary cardiac tumor diagnosis, and the importance of using multiple cardiac imaging modalities to reach an accurate diagnosis. Till now there has been no specific treatment for PCL due to the very small number of reported cases.

## Conclusions

Our case report shows the unusual presentation of ACS, rare primary cardiac tumor diagnosis, and the importance of using multiple cardiac imaging modalities to reach an accurate diagnosis. Till now there has been no specific treatment for PCL due to the very small number of reported cases. Identifying rare presentations of PCL, emphasizing its atypical mimicry of ACS. Understand the pivotal role of multimodal imaging, including echocardiography and CMR, in precise cardiac tumor diagnosis. Grasp the diagnostic complexities and limited treatment options for PCL, highlighting the aggressive nature of certain cardiac malignancies. Appreciate the educational significance of this case in raising awareness of unusual ACS presentations and rare cardiac tumors. Using multiple cardiac imaging modalities can enhance the accurate diagnoses of many unusual presentations of ACS.
